# Human interactive liquid crystal fiber arrays

**DOI:** 10.1126/sciadv.adp0421

**Published:** 2024-09-06

**Authors:** Samuël A. M. Weima, Reza Norouzikudiani, Jaeryang Baek, Jacques A. Peixoto, Thierry K. Slot, Dirk J. Broer, Antonio DeSimone, Danqing Liu

**Affiliations:** ^1^ Laboratory of Human Interactive Materials (HIM), Department of Chemical Engineering and Chemistry, Eindhoven University of Technology, Den Dolech 2, 5612 AZ Eindhoven, Netherlands.; ^2^ Institute for Complex Molecular Systems (ICMS), Eindhoven University of Technology, Den Dolech 2, 5612 AZ Eindhoven, Netherlands.; ^3^ The BioRobotics Institute, Scuola Superiore Sant’Anna, Viale Rinaldo Piaggio, 34, Pontedera 56025, Italy.; ^4^ School of Computing Science, Simon Fraser University, 8888 University Drive, Burnaby, BC V5A 1S6, Canada.; ^5^ Schulich Faculty of Chemistry, Technion–Israel Institute of Technology, Haifa 3200003, Israel.; ^6^ SISSA-Scuola Internazionale Superiore di Studi Avanzati, Trieste 34136, Italy.

## Abstract

This paper presents interactive liquid crystal fiber arrays that can actuate in a way perceptible by human touch. The fibers are actuated via a computer interface, enabling precise control over actuation direction, magnitude, and frequency. Unlike conventional methods, our technique initiates the actuation at the base of the fibers, which is enabled by fabricating the fibers directly onto an electrical circuit. Fiber actuation is achieved by localized addressing of an in situ formed radially aligned segment. This induces reduction in the scalar order parameter and leads to deformation of the fiber base, causing bending toward the activated region. Extensive modeling validates this actuation mechanism and identifies optimal conditions and actuation strategies for achieving the desired responses. The actuation process is rapid, is highly reversible, and maintains excellent performance over repeated (>200) cycles. These liquid crystal fiber arrays provide a safe contact with humans or other objects, making them highly suitable for applications in smart wearable devices and immersive interfaces.

## Teaser

The electrothermal bending of liquid crystal fibers is used to design an interactive device to allow remote haptic communication.

## INTRODUCTION

In the realm of soft robotics, substantial advancements have been made in the field of human-machine interactions, leveraging responsive materials that exhibit reversible deformation when being subjected to specific stimuli. Such adaptability has paved the way for innovative applications, particularly for those that interact with delicate objects like the human body (*1*). Among the diverse soft robotic approaches explored, liquid crystal elastomers (LCEs) (*2*, *3*) have emerged as a standout option for relying on their molecular alignment to achieve unique anisotropic deformations. For the best interaction with humans, we propose configuring the responsive materials into fiber arrays. Fiber arrays, characterized by their structured and versatile form, are integral to providing targeted control and facilitating localized motion. Liquid crystal fibers (LCFs) have demonstrated their potential as artificial muscles, tentacles, and haptic feedback systems, offering exciting possibilities for interaction and feedback in various fields. Known techniques to create LCFs include three-dimensional printing for complex geometries (*4*–*6*), (electro) spinning for very thin fibers (*7*–*10*), and drawing for freestanding fibers (*11*–*13*). While previous research has explored photomechanical (*11*, *14*, *15*) and photothermal (*16*–*18*) actuation methods for LCFs, these techniques often necessitate complex optics setups and precise light source. Moreover, morphing is the result of a deformation of the whole fiber body by an asymmetric response over the cross section of the fiber resulting in one-directional bending, which limits practical applications in human-machine and machine-machine interactions.

In our work, we introduce a concept involving the integration of responsive LCFs and LCF arrays within a device, where a pivotal control mechanism is situated at the base of each fiber. Through a localized contraction at the base, our LCFs undergo a deviation from their original upright stance. This approach grants the fiber bodies an unprecedented freedom to dynamically engage with both human interaction and the environment. We further analyze our LCFs’ actuation behavior by comparing the experimental results with finite element modeling (FEM). This established (*19*–*23*) method for simulating shape changes in LCE materials allows us to gain valuable insights into the actuation mechanism and identify optimal conditions. To precisely and conveniently actuate the LCFs, we developed a computer-material interface for digital control. This interface serves as the bridge between computational systems and the physical manipulation of these fibers, offering a seamless method for users to exert precise control over the behavior and responses of the LCFs. Through this interface, users can effortlessly input commands, leveraging the power of digital control to orchestrate intricate movements and adjustments within the responsive LCFs. Our effort marks a convergence of materials innovation, device integration, and computer interfaces within a multidisciplinary research framework. Our newly developed LCFs hold remarkable promise in shaping the future of robotics and interactive technologies, paving the way for a more intuitive and seamless integration of machines into various aspects of human life.

## RESULTS

### Concept and setup

**Fig. 1.**
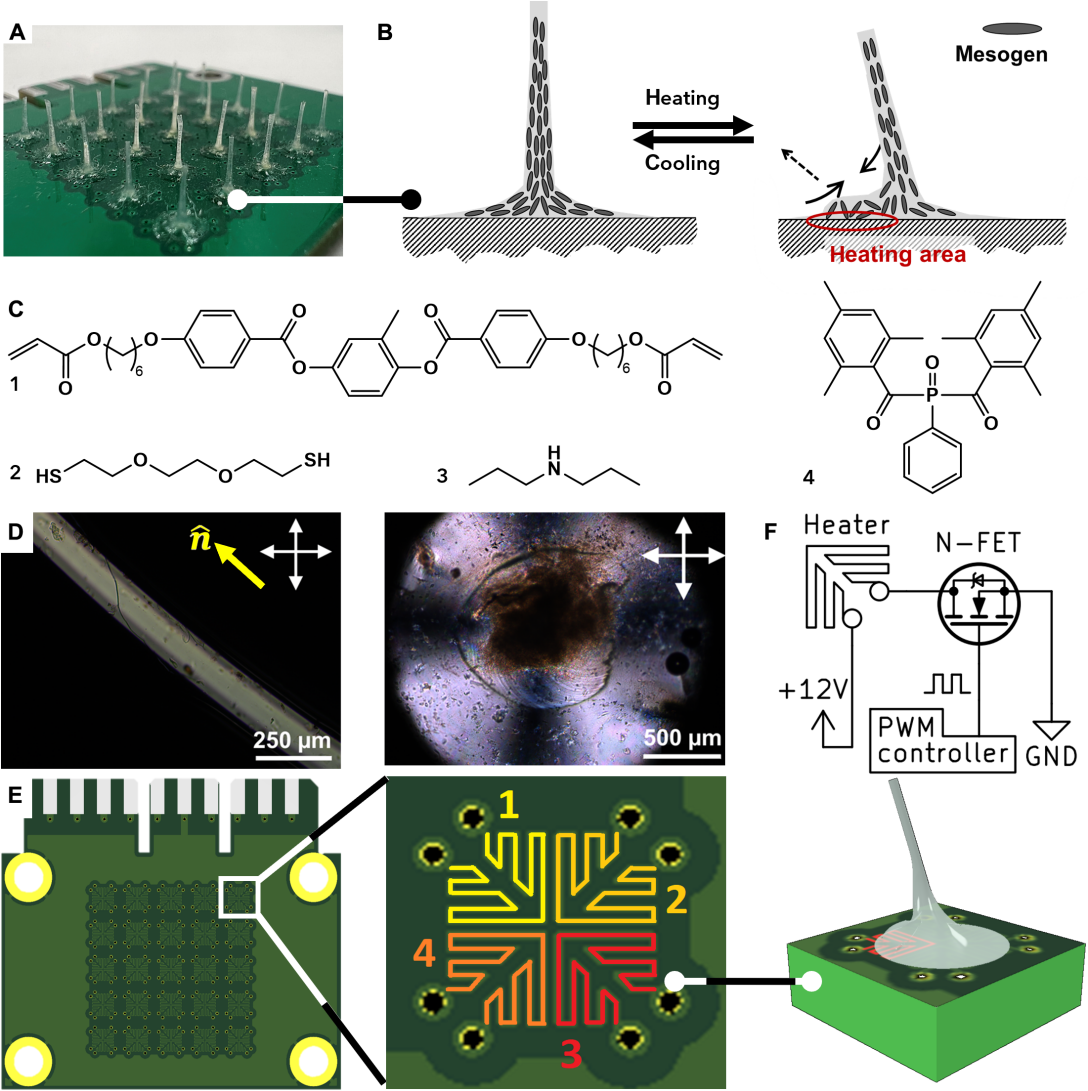
LCF actuation concept. (**A**) Array of LCFs on a PCB. (**B**) Schematic illustration of mesogen orientation and LCF actuation upon heating a section of its base. (**C**) Chemical composition for fabrication of LCFs. (**D**) POM images of the length (top) and base (bottom) of the LCF. White arrows indicate crossed polarizers, and the yellow arrow indicates alignment director. (**E**) Illustration of PCB with heating domains 1 to 4 and LCF actuation upon powering heating domain 3. (**F**) Simplified electrical scheme for a single heating domain.

Here, we present our concept for actuating LCF arrays from their base, as depicted in Fig. 1 (A and B). To achieve this goal, we developed a holistic approach, addressing various key aspects of the process. This approach includes the synthesis and optimization of liquid crystal oligomers as precursor for photopolymerization, the in situ fabrication of responsive LCFs by drawing and polymerization, the design of the electronic system that allows selective heating, and the assembly of LCFs into a cohesive system that grants precise control over their deformation.

We formulated an oligomer composition (Fig. 1C) with a 10:9 ratio of diacrylate mesogen **1** and dithiol chain extender **2**. This optimized ratio exhibits favorable rheological properties, facilitating the drawing of fibers (*11*). To prepare the oligomer, we used base catalyst **3** and subsequently combined with photoinitiator **4** for later cross-linking. To fabricate the LCFs, we developed a method of drawing from nematic polydomain oligomer on a clean surface, utilizing a combination of shear force and elongational flow to obtain mesogen alignment along the direction of flow (Fig. 1B). Subsequently, the fibers were subjected to ultraviolet (UV) light to enable the cross-linking through polymerization of the acrylate chain ends, thereby preserving the established molecular alignment within the fibers. The resulting fibers exhibit a transition from radial planar alignment at their base to a uniaxial alignment along their length toward the tip. By utilizing this approach, we successfully produced arrays of LCFs with two different fiber lengths, measuring 4 mm and 2 cm, respectively. Notably, the LCFs of both arrays exhibited nearly complete transparency, the absence of light scattering indicating a high degree of mesogen alignment with low defect rate. Even in the case of the shorter 4-mm fibers, the alignment remained impressive, despite the limited drawing distance over which alignment could be established. At the base of the fibers, the molecules are arranged in a radial manner within the plane of the base, while exhibiting splay in the cross section of the base (Fig. 1D). These distinct molecular arrangements are pivotal to the unique properties of the fibers, which will be explored for actuation purposes in this study.

The actuation of the fiber is initiated from its base. By selectively heating a specific section at the LCF base, we induce a reduction in the local order parameter. Because of the pre-established molecular alignment, this localized heating leads to contraction at the heated section, as illustrated in Fig. 1B. Consequently, the fiber undergoes deflection toward the heated section. Further increasing the local temperature above the nematic-isotropic transition temperature (*T*_ni_) at 87°C causes part of the fiber base to become isotropic. This transition causes the fiber to deflect even further from its initial position. The cross-linking ensures the stability and resilience of the fiber: After the heating stimulus is removed, the molecular alignment within the LCF is restored, causing the fiber to revert to its original shape and position.

To achieve efficient and precise heating at the base of the LCF, we opted for printed circuit boards (PCBs) as the ideal heating platform. The use of PCBs ensures controlled actuation with minimal energy loss. Furthermore, the adhesion of the fiber material to the PCB and its epoxy resin solder mask (table S1) strongly contributes to the stability during repeated actuation. The PCB design is shown in Fig. 1E. At the base of each LCF, the PCB features four domains of copper traces that can be individually heated by applying a DC voltage. As a result, a section of the LCF base will be heated and the LCF will tilt toward the heated domain.

### Actuation with constant power

**Fig. 2.**
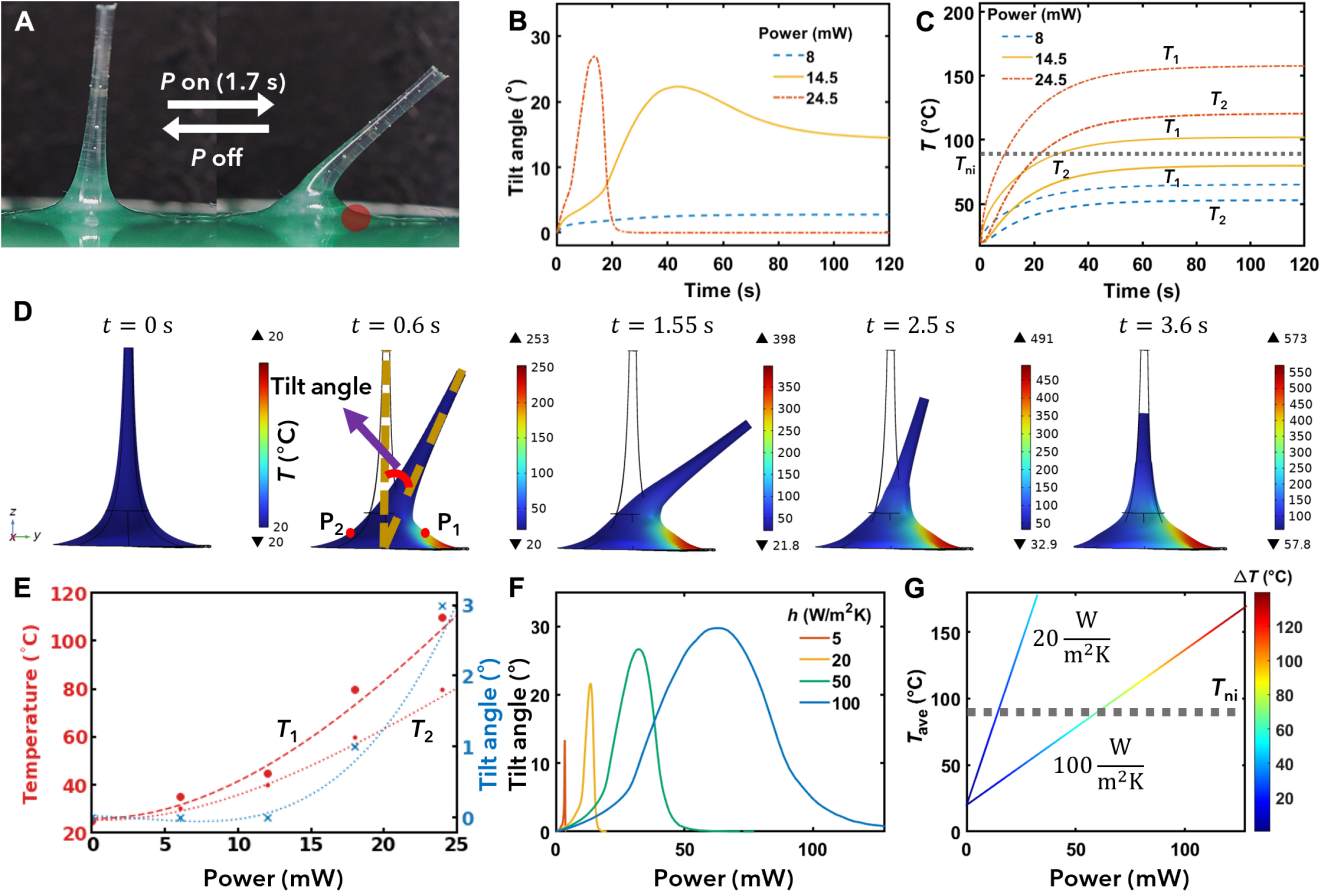
LCF actuation when supplying constant power. (**A**) Simulated tilt angle over time for different power supplies. (**B**) Simulated temperature evolution on the right (*T*_1_, corresponding to P_1_ in part D) and left of the LCF for different power supplies. (**C**) LCF actuation upon powering a single heating domain, as indicated by the red dot, for 1.7 s with 240 mW per LCF. (**D**) Simulated shape and temperature profile during bending, back-bending, and final contraction when continuing to supply 240 mW. (**E**) Experimental steady-state temperatures and tilt angle for different power supplies. (**F**) Simulated steady-state tilt angle as a function of power for various heat convection coefficients (*h*). (**G**) Simulated temperature average and difference calculated between *T*_1_ and *T*_2_ as a function of power, for different *h*.

We started our investigation on actuation with constant power by focusing on LCFs with a length of 4 mm. To change the applied power, we used pulse-width modulation, as depicted in Fig. 1F. Throughout the experiments, we found that before the transition of the base material to the isotropic state, the magnitude of bending could be controlled by adjusting the power. However, once the applied power surpassed a certain threshold, part of the fiber base underwent a transition to the isotropic phase (fig. S1). This partial transition leads to the maximum tilt angle, defined as the angle between the fiber axis and its initial axis direction. To gain deeper insights into the underlying physics governing the deformation of LCF caused by Joule heating and explore the parameters more systematically than experimentally feasible, we used FEM. See Materials and Methods for details on the used modeling technique. Our model reveals that the LCF deformation, when continuously supplied with power, could be categorized into three modes (Fig. 2, B and C).

At high power, the LCF exhibits rapid and substantial bending toward the heated region. However, the tilt angle rapidly reverts to 0 in the steady state. This reversal occurs because the temperature exceeds the nematic-isotropic transition temperature (*T*_ni_) across the entire cross section, resulting in a uniform profile of spontaneous strain. In our simulations, this actuation mode was already observed at a power of 24.5 mW, while experimentally more power was required. We attribute this mismatch to a difference between the material and environmental values used in the model and those of the experimental setup. Extensive model optimization and supporting experiments may reduce this mismatch, but this was considered unnecessary for the qualitative comparison presented here. The most substantial actuation occurred at room temperature, where a power supply of 240 mW per LCF for 1.7 s resulted in a 45° tilt of the LCF (Fig. 2A and movie S1). Using the same power in the FEM reconstruction of the experiment yields similar results (Fig. 2D), with a slight deviation in actuation speed, which can be attributed to imperfect estimations of material parameters. The model further suggests that the LCF underwent reverse bending until it eventually demonstrated a uniform contraction. It is worth noting that achieving full back-bending deformation experimentally was challenging due to the thermal degradation of both LCF and PCB at high temperatures.

At low power, such as 8 mW, the tilt angle of the LCF increases slightly over time due to the induced spontaneous strain gradient resulting from the temperature gradient along the radial direction and rise in temperature within the LCF. Eventually, the bending angle reaches its steady state due to the temperature saturation within the LCF. The corresponding experimental results are shown in fig. S2 and summarized in Fig. 2E. After conducting the power range analysis, we decided for the experiments to use 24 mW per LCF as the maximum power that could be indefinitely supplied to the fiber without any degradation. The time evolution of the temperature within the LCF is described by monitoring the temperatures *T*_1_ and *T*_2_ at specific points P_1_ and P_2_, respectively (Fig. 2D).

Simulations further indicate an intermediate mode in which the tilt angle initially increases. However in this mode, after a certain period of time, the temperature in some regions outside of the desired quadrant starts to exceed the *T*_ni_. Consequently, the isotropic region becomes less localized, resulting in a more uniform profile of spontaneous strain and leading to the back bending of the LCF toward a smaller steady-state tilt.

Next, we performed parametric studies in both experiments and model to identify conditions that could enhance the bending angle in the steady state. The heat convection coefficient (*h*) of the medium was varied, and both the tilt angle (Fig. 2F) and the average temperature (Fig. 2G) were recorded as a function of the power supply. These studies revealed that increasing the power supply initially leads to a rise in the steady-state tilt angle due to a steeper temperature gradient and due to the temperature within the LCF approaching the *T*_ni_ (Fig. 2G). However, after reaching a specific average temperature, despite the continued increase in the temperature gradient, the tilt angle decreases until the LCF totally loses its bending deformation. As shown previously, this decrease in tilt angle is caused by the temperature within the LCF exceeding *T*_ni_ in regions outside the desired quadrant, resulting in a more uniform spontaneous strain profile. In addition, our simulations revealed that a larger steady-state bending angle could be achieved by enhancing the cooling process. In practice, enhanced cooling could be most readily achieved with a thermoelectric heat exchanger combined with forced convection, potentially using fanned heatsinks. Enhanced cooling enables a larger useable temperature gradient by allowing an increase in the power supply without the average temperature exceeding the *T*_ni_.

### Actuation with periodic power

**Fig. 3.**
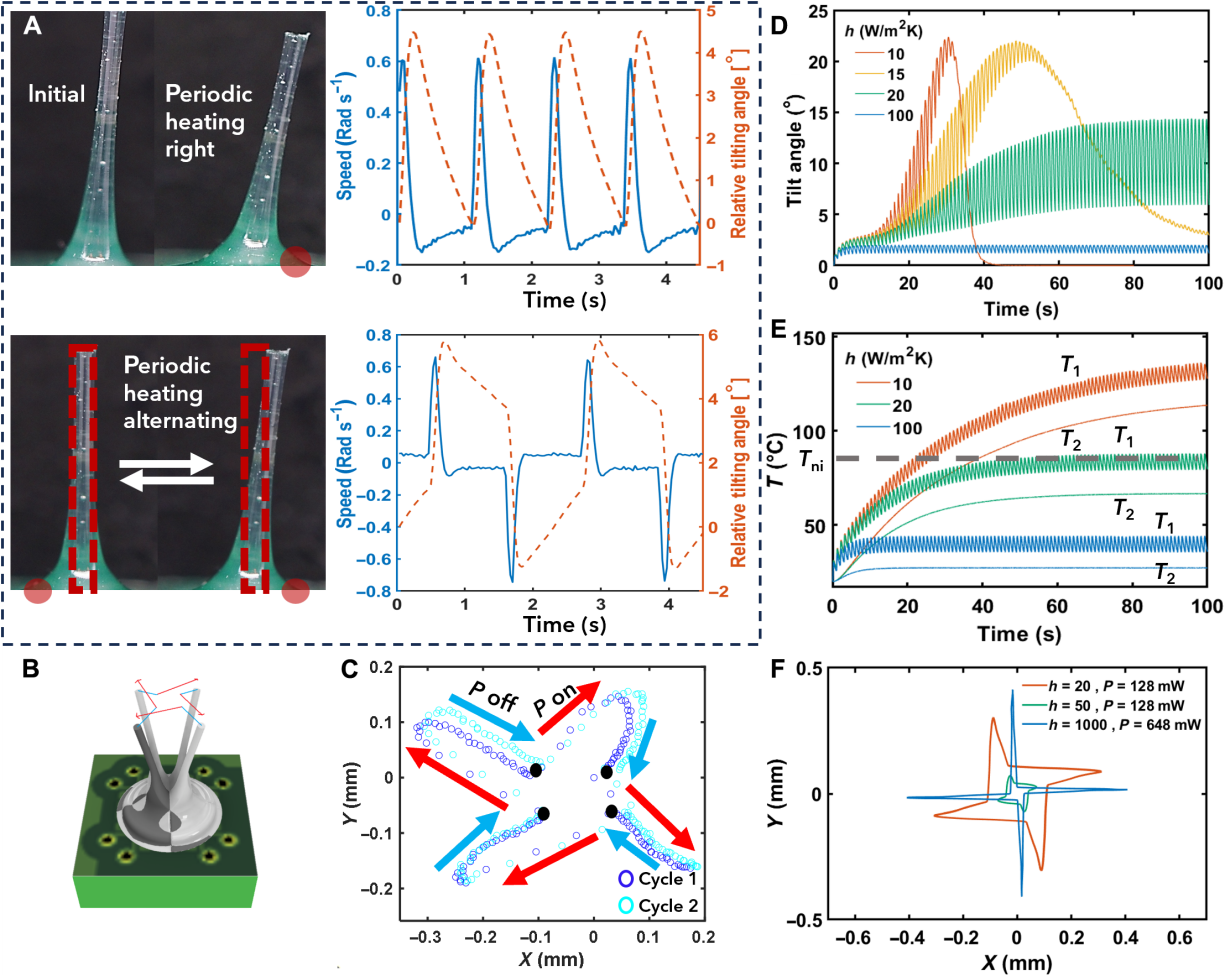
LCF periodic actuation. Power “*P*,” normalized for one LCF, equals 240 mW. This power is applied in 0.1-s pulses, each followed by 0.9 s of passive cooling time. Positive speed and angle correspond to clockwise rotation around the center of the fiber base. (**A**) Initial position and maximum tilt when applying power pulses to a single heating domain (top) or alternating between two opposite heating domains (bottom) and the corresponding angular speed and displacement (right). Dashed black lines indicate relative tilting angle of pseudorest positions. (**B**) Schematic overlay of LCF sequentially bending in four directions. (**C**) Position of LCF tip, viewed from above, traced over time when all four heating domains are powered sequentially with 240 mW. Black dots indicate pseudorest positions. (**D**) Simulated fiber bending angle as a function of time for different cooling rates and identical heating power. (**E**) Simulated temperatures *T*_1_ and *T*_2_, measured on the heated (P_1_) and nonheated (P_2_) side, respectively, as a function of time for different cooling rates and identical heating power. (**F**) Simulated path of LCF tip for different cooling and power conditions.

Our fiber can bend periodically, with a frequency matching the frequency at which a voltage pulse is applied, within a range of up to 3 Hz. Because higher frequencies lead to a reduction of the tilt angle, we maintained a frequency of 1 Hz for our standard experiments. For these experiments, we devised various power pulse patterns, all with the same overall power supply, to analyze the resulting steady-state actuation. For all patterns, we observed rapid and remarkably consistent bending of the fiber (movies S2 to S4). However, a slight bias in tilt angle may be observed, which we attribute to imperfections in fabrication. During the experiment, we observed that when multiple heating domains are sequentially heated, the LCF exhibits several pseudorest positions (Fig. 3A). This characteristic is more pronounced when heating all four heating domains in sequence (Fig. 3, B and C). We conducted a more detailed analysis of this observation using our model. In scenarios with low heat convection coefficients, the LCF exhibits an initial bending toward the heated area, coupled with an oscillating motion caused by the temperature gradient oscillation and rising temperature within the LCF. However, over time, both the LCF’s oscillation and bending angle gradually attenuate until the LCF eventually settles into a uniform contraction (Fig. 3D). The reason for this phenomenon is that the temperature within the sample surpasses the *T*_ni_ threshold, leading to a uniform spontaneous strain gradient along the radial direction. This phenomenon can be mitigated via enhanced cooling, allowing the LCF to oscillate about an equilibrium position with a constant amplitude in the steady state, driven by the temperature gradient oscillation below or around *T*_ni_ (Fig. 3E). However, once the heat convection coefficient exceeds a specific threshold, the oscillation amplitude and equilibrium position start to decrease due to the reduction in temperature gradient and overall temperature within the LCF.

We analyzed the fiber’s motion from a top-down perspective. The results presented in Fig. 3C indicate that sequential powering of different heating domains enabled controlled motion in two directions in a reproducible manner, though some asymmetry in tip displacement was observed. This asymmetry can be attributed to fabrication imperfections, causing the resting position of the LCF to deviate slightly from being perfectly perpendicular to the substrate. We further considered the impact of pulse energy and cooling rate on the actuation behavior. We experimentally varied pulse energy by adjusting the pulse length (as shown in fig. S3). The most favorable outcome, achieving a maximum tip displacement from the center of 0.3 mm, was obtained with a pulse length of 0.1 s. The associated tip speed was measured at approximately 2.5 mm/s for the heating process and around 0.2 mm/s for the cooling process in air at room temperature. It is worth noting that the relaxation of the fiber is primarily governed by the heat dissipation of the system, making it a considerably slower process compared to the actuation. We have conducted simulations to analyze the steady-state trajectory of the LCF tip in the *xy* plane with various power supplies and heat convection coefficients. The outcomes of these simulations are illustrated in Fig. 3F. The obtained deformation trend from the simulations shows reasonable agreement with the experimental observations. Similar to the experimental findings, the simulations indicate that the LCF tip does not pass its initial position during the oscillation due to the presence of a residual temperature gradient within the material. As illustrated, elevating the heat convection coefficient results in a lower residual temperature gradient during the cooling phase, causing the LCF tip to move closer to its initial position. However, this decrease in residual temperature also leads to a reduction in the oscillation amplitude. To minimize the residual temperature while maintaining the same oscillation amplitude, it is necessary to simultaneously increase both the power supply and cooling rate. Alternatively, the LCF tip trajectory could be made to approach a square shape, by decreasing the cooling time (fig. S4).

### Digital integration and human interaction

**Fig. 4.**
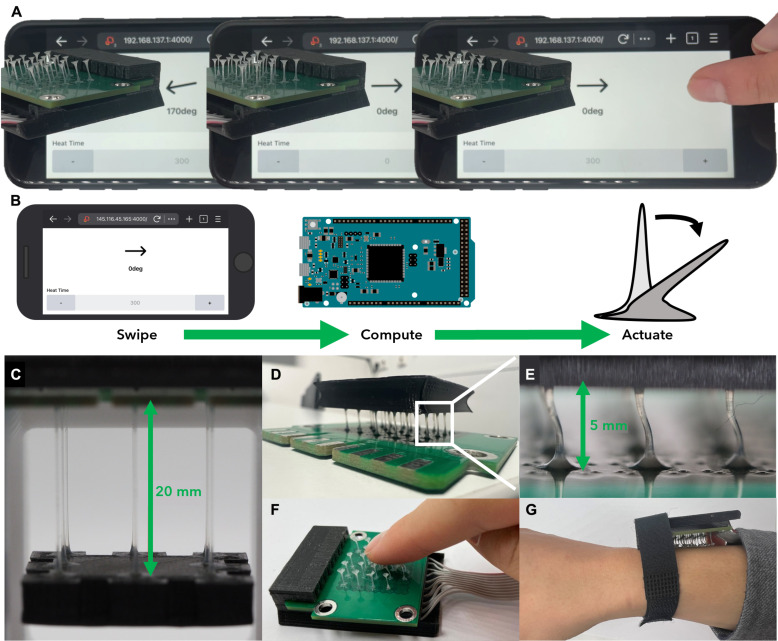
Digital and human LCF interface. (**A**) Center: LCF array at rest position when not supplying power. Left and right: Snapshots of LCF array periodically bending in the direction swiped by the user, for a heat time of 300 ms. (**B**) Schematic representation of signal-to-output pathway from phone to fiber. (**C**) Rotatable and liftable platform suspended from 20-mm-long fibers. (**D** and **E**) Array of 5-mm-long LCFs supporting a platform. (**F**) Active user interaction with LCF array. (**G**) Wearable prototype of LCF array brushing a user’s arm.

We developed a smartphone interface for controlling the LCFs. Our implementation involves a straightforward yet effective application that bridges the gap between a user’s touch input on their phone and the corresponding physical response of the LCF array. We developed a web-based user interface that captures the user’s touch interactions, such as swipes in all directions, to convey intended patterns (Fig. 4A and movie S5). The full system, from touch to motion, is illustrated in Fig. 4B.

Beyond the visual feedback, this system also allows for remote controlled displacement of objects such as an attached platform (Fig. 4, C to E, and movie S6). Depending on the layout of the PCB, concerted bending of the LCFs allowed for rotational or linear movement of the platform, effectively displacing up to roughly 180 times their own mass. In addition, the platform could be lifted via thermal contraction of the LCFs. Furthermore, we envision that a different PCB layout and actuation pattern would allow for the free transport of light objects.

Another promising application involves using the fibers to directly touch the human skin. This application could take the form of a dynamic surface for the user to touch (Fig. 4F) or the form of a wearable that touches the user (Fig. 4G). Using their smartphones, users could remotely interact via fiber arrays to replicate the intricate feel of human touch. Furthermore, by integrating our fibers with virtual reality/augmented reality systems, we could provide users with an immersive sensory experience that includes the sensation of touch, thereby substantially advancing the realism of virtual interactions.

## DISCUSSION

In conclusion, we have developed interactive LCF arrays that are directly integrated on a PCB with built-in heating domains. By contracting their radially aligned base, the body of the fiber is deflected from the original upright position. We studied both continuous and periodic modes of actuation. The maximum magnitude of actuation was a relative tilt of 37°, rapidly obtained when continuously supplying power. Eventually, when the LCF becomes saturated with heat, a uniform contraction along the fiber direction occurs. Control over periodic actuation was also demonstrated, with the frequency, magnitude and direction of actuation being dependent on the frequency, power, and pattern of the applied electrical pulses. An actuation frequency of 1 Hz was readily obtained and can still be obtained after a year of experimental use, without any observable decrease in magnitude of actuation. At this frequency, the maximum sustainable magnitude of actuation was a relative tilt of 4° observed at a peak temperature of 160°C (Δ*T* = 60°C). Prior work suggests that this actuation temperature can be reduced with a different oligomer composition (*24*). In addition, simulations revealed that sharper actuation could be achieved with a combination of high power and high cooling. We further integrated the materials into a device where a smartphone interface was developed to demonstrate remote control of the LCF array. Such remote control is expected to be useful for many applications, but of particular importance to remote haptic interactions.

Overall, the LCFs studied here have proven safe, tough, producible in bulk, and actuatable with a large magnitude and high frequency. We anticipate the developed responsive fiber arrays find a wide range of applications ranging from soft robotics, haptics to wearable devices.

## MATERIALS AND METHODS

### Material suppliers

Diacrylate mesogen 1,4-bis-[4-(6-acryloyloxyhexyloxy) benzoyloxy]-2-methylbenzene (RM82) was synthesized by Philips. Dithiol chain extender ethylenedioxydiethanethiol 95% (DODT) and catalyst dipropylamine 99% (DPA) were purchased at Sigma-Aldrich. Dichloromethane 99.9% (DCM) was purchased at Biosolve (ref 137905). Photoinitiator Irgacure819 was purchased at BASF.

### Oligomer preparation

RM82 (10 g), DODT (2.45 g), and DCM (20 ml) were combined in a flask and continuously stirred. Once a homogeneous solution was obtained, DPA (five drops) was added. The mixture was subsequently stirred overnight at room temperature. Irgacure819 (0.2 g) was added and the mixture was stirred until a homogeneous solution was obtained. DCM was evaporated via a rotary evaporator at 40°C for 30 min at 500 mbar and 30 min at 10 mbar, and via vacuum oil pump for 1 hour.

The oligomer was analyzed via NMR, GPC, and DSC (fig. S5, A to C), revealing an average chain length of 9.5 RM82 units, a polydispersity index of 2.4 and a nematic region from 46° to 87°C.

### LCF fabrication

**Fig. 5.**
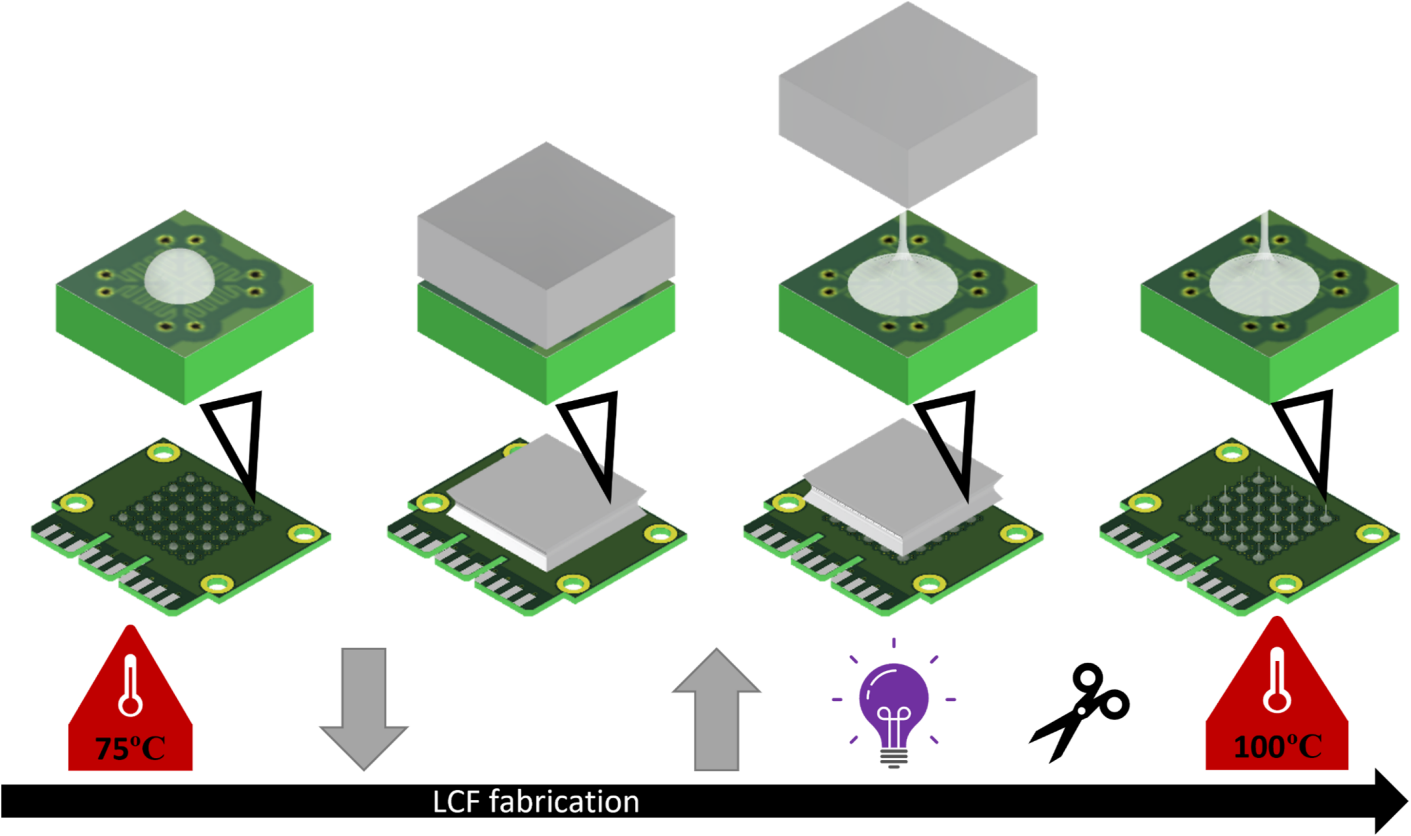
LCF fabrication.

Pieces of oligomer (3 mg) were placed on the PCB, which was then heated to 75°C to make the oligomer sufficiently fluid without exceeding the nematic-isotropic transition temperature (fig. S5D). Once the pieces had morphed into hemispheres, the PCB was pressed against a counter-plate that had also been heated to 75°C, with a spacing of 0.2 mm. After pressing for 1 s, PCB and the counter-plate were pulled apart, thereby drawing LCFs of a desired length. For both pressing and drawing, the maximum speed was 5 mm/s and the acceleration and deceleration were 5 mm/s^2^. The LCFs were subsequently UV–cross-linked (470 nm) under nitrogen atmosphere to prevent oxygen inhibition. Afterward, the LCFs could be cut if desired and lastly heated to 100°C in an oven to complete cross-linking. The entire fabrication process was performed in a regular polymer laboratory. The complete LCF fabrication process is illustrated in Fig. 5.

### Analytical equipment

Mesogen alignment was determined via crossed polarizer integrated in an optical microscope (Leica DM2700). The nematic region of the oligomer was determined via differential scanning calorimetry (DSC Q2000). Number average oligomer chain length was determined via nuclear magnetic resonance (Bruker 400B). Polydispersity index was determined via gel permeation chromatography (Shimadzu LCsolution). Complex viscosity was determined via rheometry (AR-G2 Magnetic Bearing Rheometer). LCF mechanical properties were determined via dynamic mechanical analysis (DMA Q800).

### Image analysis

A custom MATLAB script was used to track the fiber. The initial video was turned into a black and white series of images based on the YCrBr pixel values. A threshold based on the Y values was used as a criterion. The second step was to use the RegionProps function to track the center of mass of the binary images. The position of the center of the tracker was used to calculate the angle or/and the position of the fiber by direct comparison with the resting position based on simple geometric calculations.

### Finite element modeling

**Fig. 6.**
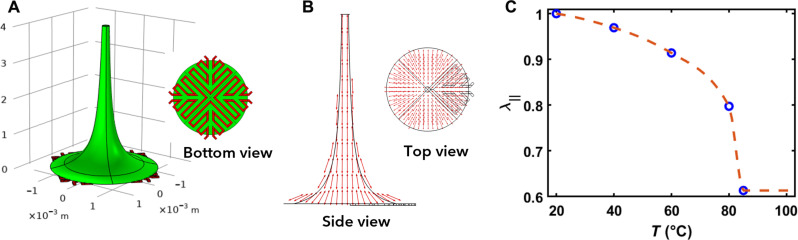
Modeling assumptions. (**A**) Geometry of LCF and heating circuit. (**B**) Alignment director following the curve of the LCF. (**C**) Measured (dots) and interpolated (dashed line) spontaneous deformation parallel to LC alignment as a function of temperature.

We developed a three-dimensional multi-physics model consisting of one LCF and its associated electric circuits in COMSOL Multiphysics (Fig. 6A). In constructing the model, we used experimental data and made certain assumptions. We represented the outer surface’s shape of each LCF by a two-dimensional hyperbolic curve ($\frac{\alpha }{r}+\beta$) revolving around the *z* axis. In this equation, *r* represented the radial distance from the *z* axis, and constants α and β were determined based on the bottom and top radius values. We also assumed that the LCF was in the nematic state in the reference configuration, with directors tangent to the outer surface and becoming more vertical as they approached the *z* axis (Fig. 6B). To simulate the LCF deformation under Joule heating, we considered three different coupled physics. First, we used the conservation of currents to calculate the electric potential distribution (*V*) in the electric circuit under applied voltage difference at the boundaries

$$-\nabla \cdot \left(\sigma \nabla V\right)=0$$ (1)

where σ is the electrical conductivity of the circuit. The applied voltage induced a current that flowed through the circuit and generated heat in the circuit, which could be described by

$$Q_{\mathrm{ec}}=\sigma {\left|\nabla V\right|}^{2}$$ (2)

We then computed the temperature (*T*) distribution in both LCF and circuit using the heat equation, taking into account the generated heat (*Q_ec_*) within the electric circuit

$$\rho C_{p}\frac{\partial T}{\partial t}-\nabla \cdot \left(k\nabla T\right)=Q_{\mathrm{ec}}$$ (3)

where ρ is the density, *C_p_* is the heat capacity, and **k** is the thermal conductivity tensor. The heat generation in the circuit increased the temperature of the entire structure leading to a reduction in the LCF order parameter and causing spontaneous deformations in both parallel (λ_‖_) and perpendicular (λ_⊥_) directions to the LC’s alignment (**n**). Last, the LCF displacement (*u*, *v*, *w*) due to the induced spontaneous stretches was computed using the mechanical equilibrium equation for the LCF

∇·S=0 (4)

where **S** is the first Piola-Kirchhoff stress tensor defined by the derivative of elastic energy (*W_el_*) with respect to the deformation gradient tensor (**F**)

$$S=\frac{\partial W_{\mathrm{el}}}{\partial F}$$ (5)

We assumed that the elastic energy (*W_el_*) of the nematic LCF is governed by the incompressible Neo-Hookean model

$$W_{\mathrm{el}}=\frac{\mu }{2}\left(I_{1}\left(F_{\mathrm{el}}^{T}F_{\mathrm{el}}\right)-3\right)-p\left(\text{det}\left(F_{\mathrm{el}}\right)-1\right)$$ (6)

where μ is the shear modulus and *p* is the Lagrange multiplier to ensure the incompressibility of the LCF. Moreover, **F***_el_* is the elastic deformation gradient tensor, which is described by

$$F_{\mathrm{el}}=FF_{s}^{-1}$$ (7)

In Eq. 7, **F***_s_* is the spontaneous deformation gradient tensor with respect to the nematic state

$${\left(F_{s}\right)}_{\mathrm{ij}}=\lambda_{\|}n_{i}n_{j}+\lambda_{⊥}\left(\delta_{\mathrm{ij}}-n_{i}n_{j}\right)$$ (8)

where δ*_ij_* is the Kronecker delta, δ*_ij_* = 1 if *i* = *j* and δ*_ij_* = 0 if *i* ≠ *j*. The value of λ_‖_ was obtained experimentally for certain temperatures and interpolated for others (Fig. 6C). In addition, λ_⊥_ was calculated considering the incompressibility of the LCF ($\lambda_{\|}\lambda_{⊥}^{2}=1$). Moreover, we considered clamped boundary condition at the base of the LCF for the mechanical equilibrium equation, and heat convection boundary condition for heat equation.

The coupled governing equations (Eqs. 1, 3, and 4) were implemented into COMSOL Multiphysics through the weak form PDE interface to obtain unknowns (*u*, *v*, *w*, *V*, *T*). The implicit adaptive step-size BDF solver was used for the time-stepping. A quasi-Newton algorithm was used to iteratively solve the nonlinear algebraic system resulting from the finite element discretization at each time step. The multifrontal massively parallel sparse direct solver (MUMPS) was chosen for the solution of the linearized system at each iteration. A mesh convergence study was also implemented to ensure that the results were independent of the mesh size.

For the simulations conducted, the material parameters were either extracted from previous papers or obtained by fitting the simulations to experimental results. Because of the anisotropic heat conduction within the LCEs (*25*), the heat conductivity parallel (*k*_‖_) and perpendicular (*k*_⊥_) to the LCs’ alignment direction were assumed to be 1.5 W m^−1^K^−1^ and 0.2 W m^−1^ K^−1^, respectively. The thermal diffusivity (*k*_⊥_/ρ*C_p_*) of the LCF perpendicular to the alignments’ direction was considered to be 1 × 10^−7^m^2^ s^−1^ (*26*). The Young’s modulus (*E*) of the LCF was measured experimentally (fig. S5E) and found to be 40 MPa in room temperature. In the simulations, for simplicity, the temperature dependency of Young’s modulus was neglected. The heat convection coefficient (*h*) for natural convection in air was determined to be 20 W m^−2^ K^−1^ through comparisons between the simulations and experimental observations. Furthermore, the electric circuit used in the experiment was constructed using copper as the material with a thermal conductivity of 400 W m^−1^ K^−1^, a thermal diffusivity of 1.16 × 10^−4^ m^2^s^−1^, and an electrical conductivity of 59.7 × 10^6^ S m^−1^.

### Electronics design

The PCB shown in this paper featured 25 heating units arranged with grid symmetry. Each heating unit consisted of four heating domains and was made to support one LCF. When viewed from the top, this layout resulted in concerted LCF actuation toward any corner of the grid, depending on which heating domains were powered. The design of this PCB, in addition to the one used for the rotation of a platform and the one used for the power controller, can be found in fig. S6. Please contact the authors for any further clarification on the workings of the controller or its associated Arduino code (*27*).

### Smartphone interface

To enable remote control of the fiber array, we developed a web-based user interface using SvelteKit, a modern frontend framework we chose for its efficiency and lightweight builds. The user interface allows the user to swipe in the direction they wish the fibers to bend, and tap to change the magnitude of bending.

The backend, coded in Python, serves two main functions: hosting the web server and handling serial communication with the Arduino microcontroller. The real-time aspect of the system is achieved using WebSocket, a web technology protocol that facilitates interactive communication sessions between the user’s browser and the server. This allows for the immediate transmission of touch data from the phone interface to the backend system.

Upon receiving data from the WebSocket connection, the backend translates these inputs into specific commands for the Arduino microcontroller. These commands generate the desired patterns of thermal-induced actuation of the fiber array, allowing for actuation that closely mimics the input touch. The full code can be found on Zenodo (*27*).
