# Supplementary material for: Human interactive liquid crystal fiber arrays
Source: Sci Adv. 2024 Sep 6;10(36):eadp0421. doi: 10.1126/sciadv.adp0421 (PMC11378903; doi:10.1126/sciadv.adp0421)
Supplement: Supplementary file 1 — Figs. S1 to S6 Table S1 Legends for movies S1 to S6 [file sciadv.adp0421_sm.pdf]

Science Advances
AAAS

Supplementary Materials for

Human interactive liquid crystal fiber arrays

Samuël A. M. Weima *et al.*

Corresponding author: Danqing Liu, d.liu1@tue.nl

Sci. Adv. **10**, eadp0421 (2024)
DOI: 10.1126/sciadv.adp0421

The PDF file includes:

Figs. S1 to S6
Table S1
Legends for movies S1 to S6

Other Supplementary Material for this manuscript includes the following:

Movies S1 to S6

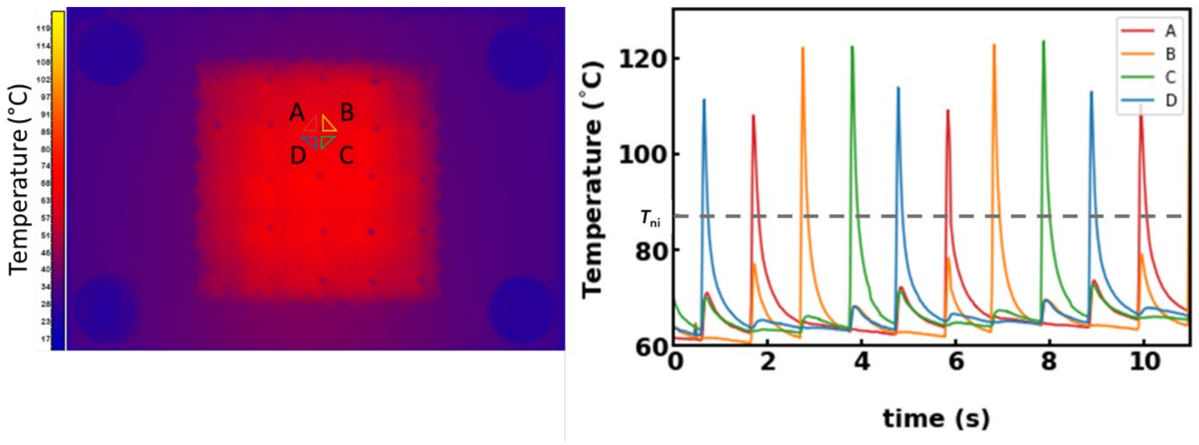

Fig. S1. Temperature evolution of four sections of LCF base.

Left: top-view thermal image of PCB with four heating domains A-D indicated with colored triangles. Right: maximum temperature measured for heating domains A-D, when applying 240 mW per LCF in 0.1 s pulses, each followed by 0.9 s of passive cooling time. The pulses iterate over the heating domains in clockwise fashion.

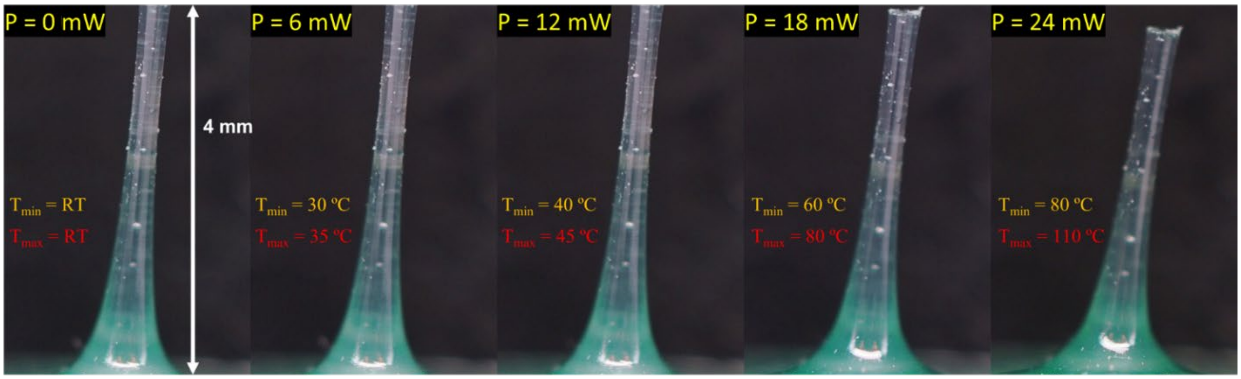

Fig. S2. LCF shrinking and tilting under the continuous application of power to one heating domain.

Power *P* is normalized for one fiber. T_{min} corresponds to the temperature of the heating domain opposite to the one being powered. T_{max} corresponds to the temperature of the heating domain being powered. The images correspond to the equilibrium positions of the fiber for a set power, in ambient air.

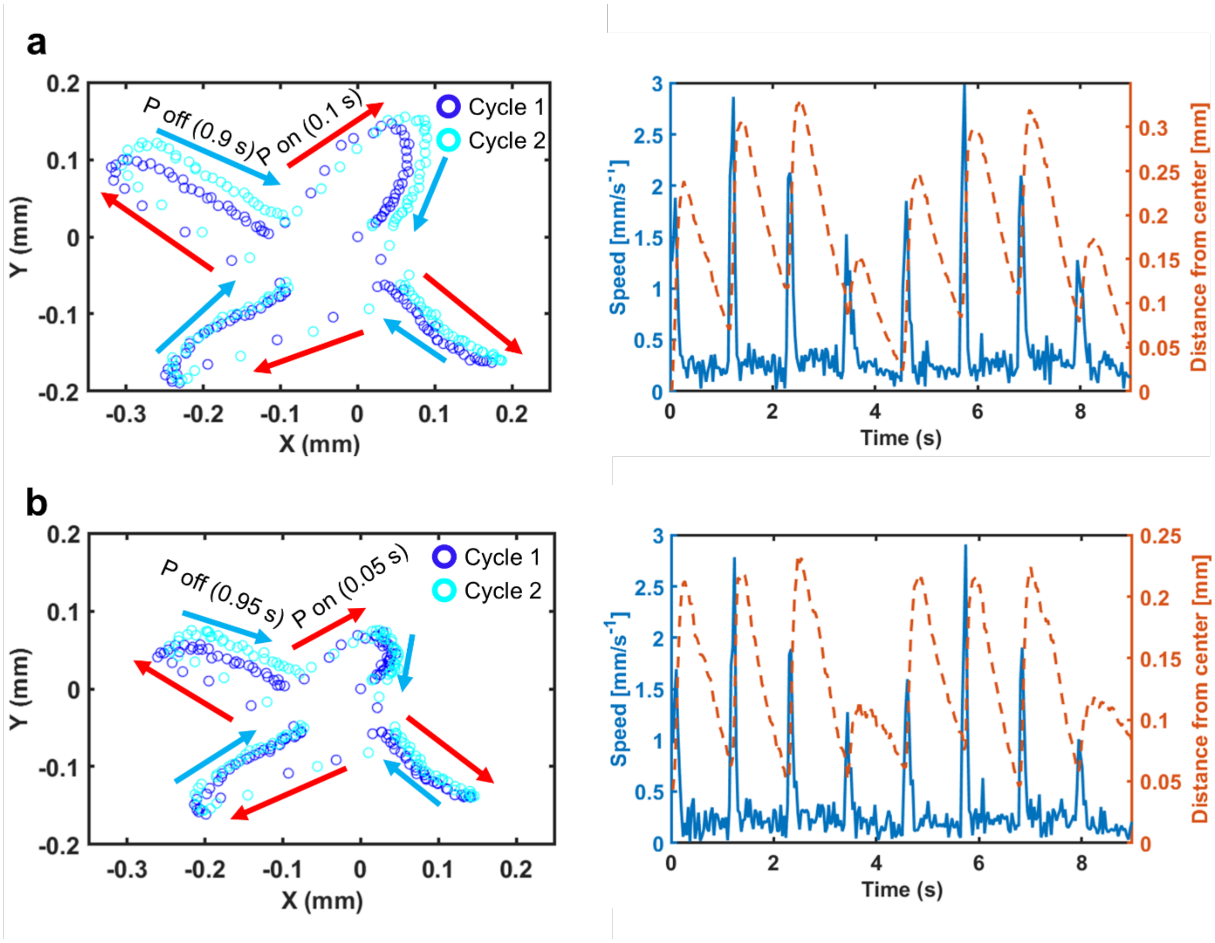

Fig. S3. LCF tip displacement for different pulse lengths.

LCF tip displacement path (left), speed and distance (right) for a pulse length of (**a**) 0.1 s and (**b**) 0.05 s. Power *P* equals 240 mW per LCF.

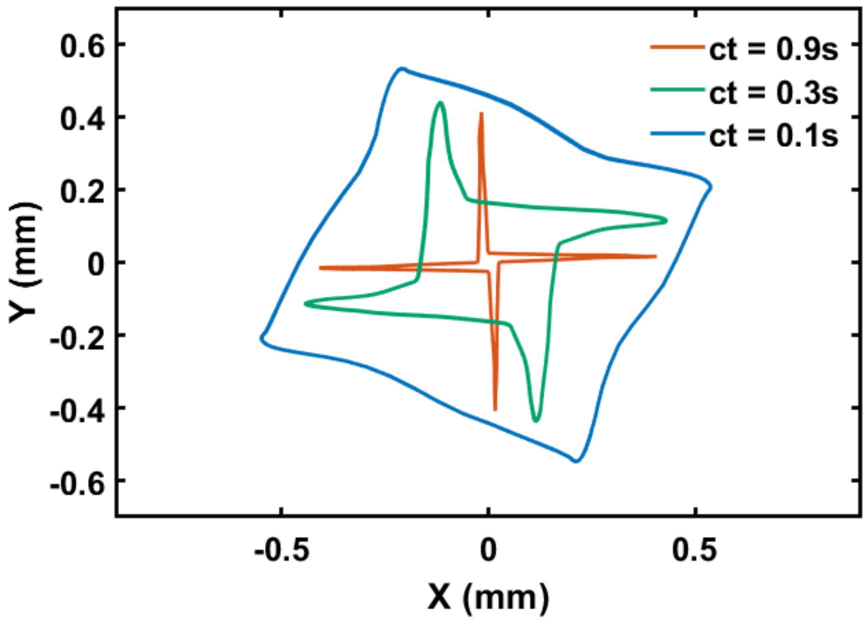

Fig. S4. Simulated LCF tip trajectories for different cooling times (ct).

Power P equals 648 mW and heat convection coefficient equals 1000 $Wm^{-2}K^{-1}$.

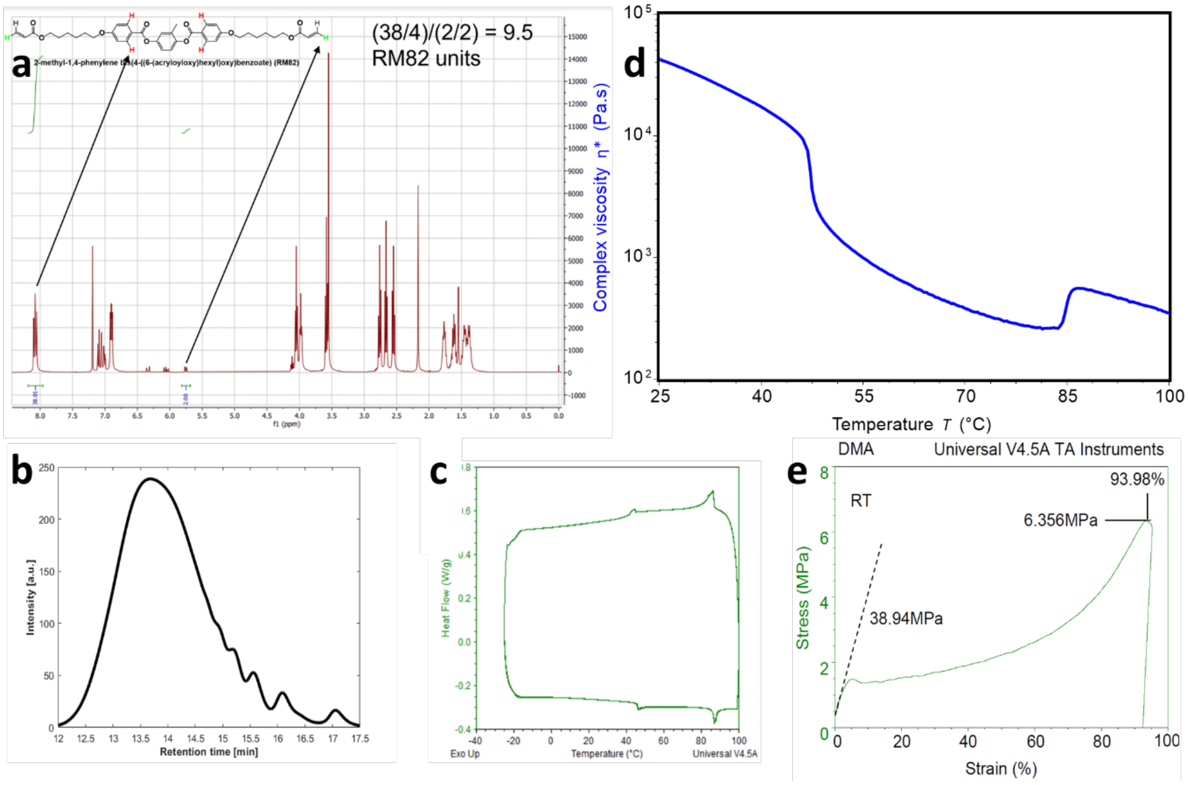

Fig. S5. Material analysis.

(**a**) ^{1}H-NMR spectrum of oligomer. (**b**) Gel Permeation Chromatograph of oligomer. (**c**) Differential Scanning Calorimetry of oligomer. (**d**) Complex viscosity of oligomer as a function of temperature, measured with parallel plates. (**e**) Dynamic Mechanical Analysis of LCF at room temperature using Displacement Ramp.

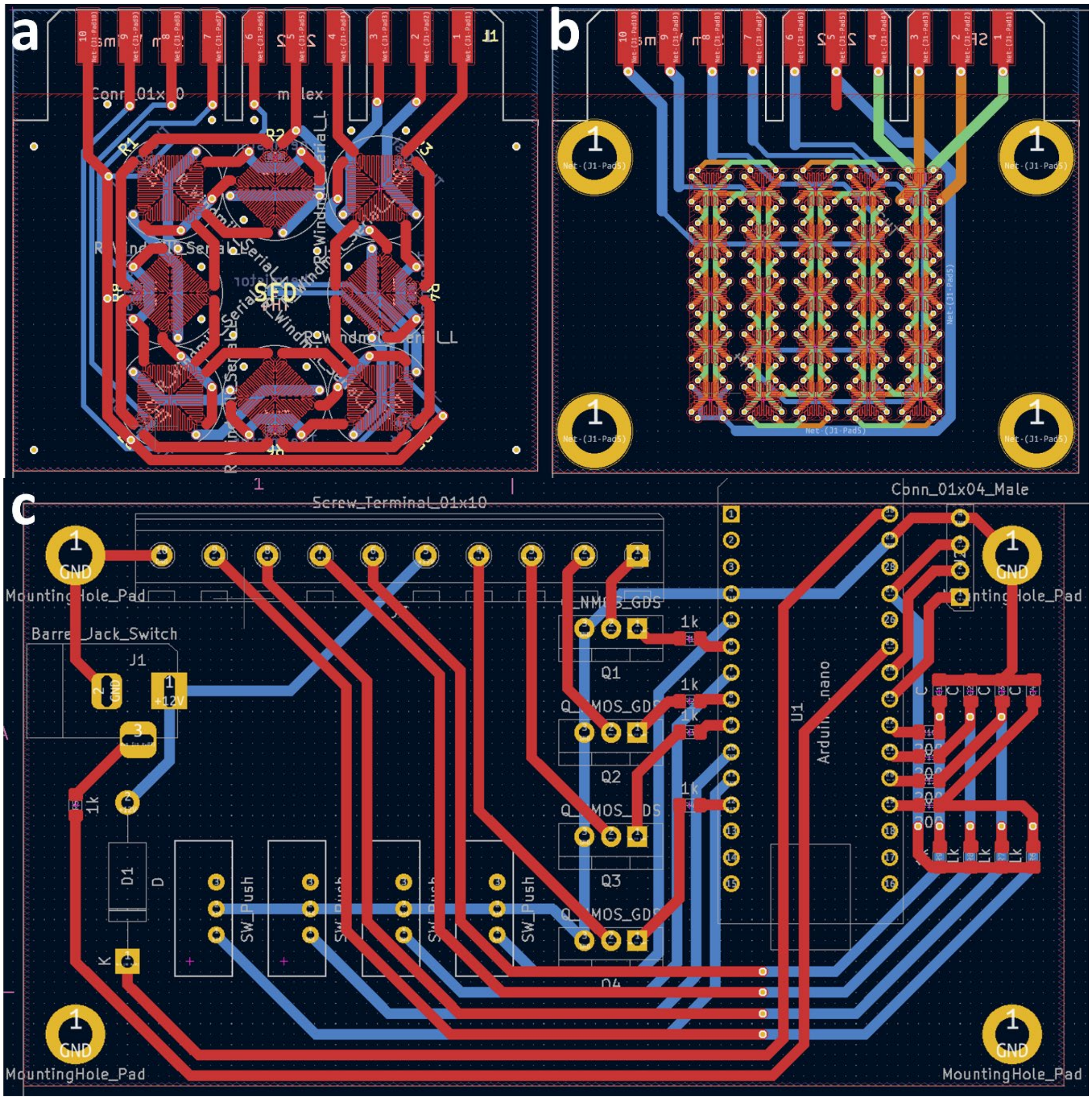

Fig. S6. PCB designs.

Designs for (**a**) PCB used for LCF driven rotational motion of a platform, (**b**) PCB used for all other actuation described in this paper, (**c**) controller used to direct power to the PCBs.

Table S1. PCB solder mask composition.

PCBs manufactured by PCBWay, https://www.pcbway.com/

Compound	CAS NO.	wt%
Poly(Bisphenol A-co-epichlorohydrin)	9003-36-5	17
Poly[(o-cresyl glycidyl ether)-co-formaldehyde]	29690-82-2	26
Dipentaerythritol hexaacrylate	29570-58-9	10
Barium sulfate	7727-43-7	20
Silicone oil	63148-62-9	2
Silicon dioxide	7631-86-9	2
Copper(II) phthalocyanine	147-14-8	1
DBE dibasic ester	95481-62-2	22

Movie S1. Maximum thermal actuation of liquid crystal fiber.

The most substantial actuation occurred at room temperature, where a power supply of 240 mW per LCF for 1.7 seconds resulted in a 45-degree tilt of the LCF.

Movie S2. Liquid crystal fiber bending periodically in one direction.

One heating domain is being powered using 188 mW per LCF with 100 ms pulses, at a frequency of 1 Hz.

Movie S3. Liquid crystal fiber bending periodically in two directions.

Two diagonally opposed heating domains are being powered alternatingly using 188 mW per LCF with 100 ms pulses, at a combined frequency of 1 Hz.

Movie S4. Liquid crystal fiber bending periodically in four directions.

Four heating domains are being powered sequentially in a clockwise fashion using 188 mW per LCF with 100 ms pulses, at a combined frequency of 1 Hz.

Movie S5. Liquid crystal fiber bending controlled via smartphone interface.

A grid of LCFs periodically bend towards the direction dictated by the direction in which a user swipes. Intensity of bending is controlled via the length of the pulses, and the user can change this length by pressing buttons on the screen.

Movie S6. Liquid crystal fibers suspending and rotating a platform.

Eight LCFs with a length of 2 cm suspend a platform weighing 4 g and rotate it due to power pulses being applied to heating domains in a clockwise fashion, when looking at the PCB as in Fig. S6A, at a frequency of 1 Hz.
